# An *E*/*Z* conformational behaviour study on the trypanocidal action of lipophilic spiro carbocyclic 2,6-diketopiperazine-1-acetohydroxamic acids

**DOI:** 10.1016/j.tetlet.2013.03.128

**Published:** 2013-06-19

**Authors:** Alexandra Tsatsaroni, Grigoris Zoidis, Panagiotis Zoumpoulakis, Andrew Tsotinis, Martin C. Taylor, John M. Kelly, George Fytas

**Affiliations:** a Faculty of Pharmacy, Department of Pharmaceutical Chemistry, University of Athens*,* Panepistimioupoli-Zografou, GR-15771 Athens, Greece; b Institute of Biology, Medicinal Chemistry and Biotechnology, National Hellenic Research Foundation, 48 Vas. Constantinou Ave., 11635 Athens, Greece; c Department of Pathogen Molecular Biology, London School of Hygiene and Tropical Medicine, Keppel Street, London WC1E 7HT, UK

**Keywords:** Lipophilic hydroxamic acid derivatives, *E*/*Z* conformational behaviour, Trypanocidal action, NMR, Molecular modelling studies

## Abstract

An explanation for the vast difference observed in the trypanocidal activity between the new secondary (*N*-methylated) hydroxamic acids **5** and **6**, and their primary (nonmethylated) congeners **1a** and **2**, based on their *E*/*Z* conformational behaviour in DMSO, is presented.

## Graphical abstract

**Figure:**
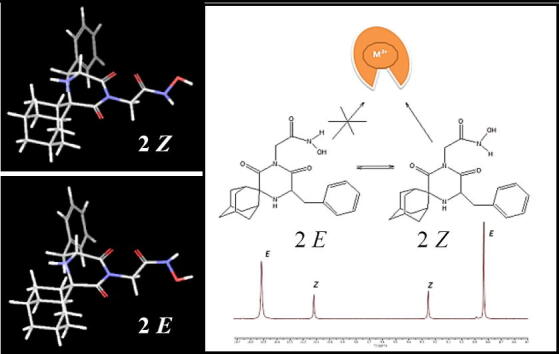

Primary and secondary hydroxamic acids [R′CON(R)OH, R = H or alkyl] constitute a class of metal ion chelating agents with great therapeutic potential.^1^, (a) A large number of naturally occurring hydroxamate-based siderophores (Fe^3+^ carriers) have been isolated and much effort has been devoted to the synthesis of bioactive hydroxamic acids.(b), 2, (a), (b) Due to their metal ion binding ability, hydroxamic acids often behave as inhibitors of metalloenzymes (e.g., Fe^3+^-containing lipoxygenase, Zn^2+^-containing MMPs and HDACs and Ni^2+^-containing urease), which are implicated in the pathophysiology of human diseases.(a), (a), 3

The structures of hydroxamic acid analogues have been studied extensively using NMR and molecular modelling techniques.^4^, ^5^ For particular hydroxamic acid structures, the existence of different possible conformations in solution has been found to depend on the sample concentration, the temperature and the solvent.^6^ More specifically, the hydroxamate group may adopt *E* and *Z* conformations which are separated by a high energy barrier.^7^ Furthermore, tautomerism between the amide and the imide forms is possible, but the imide forms are found to be absent in solution.^7^ Various researchers have concluded that the *Z* conformation of the amide structure prevails since it becomes stabilized via hydrogen bond formation, either intramolecularly or intermolecularly to a polar solvent.^7^ Interestingly, in some cases, the proton NMR spectra of hydroxamic acid analogues at room temperature (∼25 °C) clearly show a double set of characteristic peaks, arising from the two different isomers (*E*/*Z*), while in others, a single set of characteristic peaks is observed, which is attributed to one of the two isomers. Thus far, the most extensive conformational study reported refers to benzohydroxamic acid (BHA) in acetone.^7^ Moreover, it is well-known that hydroxamic acids, R′CON(R)OH (R = H or alkyl), chelate with a variety of metal ions via the oxygen atoms, the carbonyl oxygen and the deprotonated OH (*O*,*O*-co-ordination). In this well documented *O*,*O*-type co-ordination mode, the amide structure of the hydroxamic group must adopt the *Z*(*cis*) conformation (ion-binding conformation) for effective ion complexation.^8^

In a previous publication, we reported on acetohydroxamic acid analogues, derived from conformationally constrained lipophilic spiro carbocyclic 2,6-diketopiperazine scaffolds by attaching the acetohydroxamic acid group to their imide nitrogen, as a potential metal ion complexing group. The primary hydroxamic acid derivatives **1a**–**e**, **2**, **3a**–**d**, **4a** and **4b** (Fig. 1) exhibited excellent trypanocidal properties.9a We have also demonstrated that the hydroxamic acid group (CONHOH) is a requirement for activity. On this basis, we assumed that this class of compounds acts by inhibiting a vital parasite metalloenzyme, through metal ion binding at this group.

**Figure 1:**
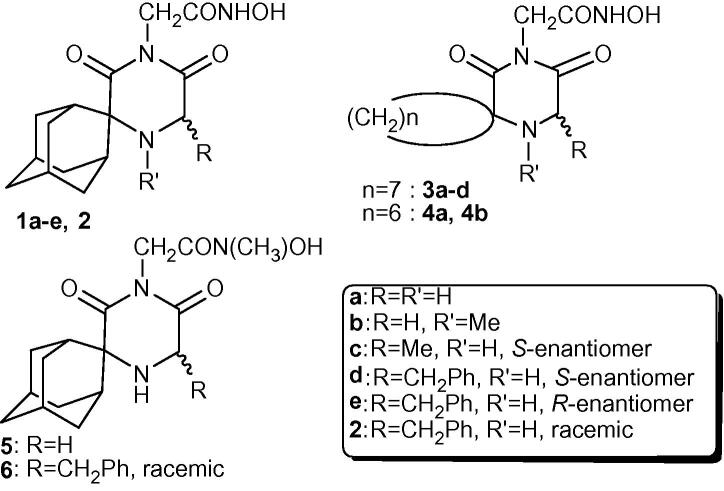
Structures of the hydroxamic acid derivatives **1a**–**e**, **2**, **3a**–**d**, **4a** and **4b** and the structures of the new *N*-methyl hydroxamate analogues **5** and **6**.

As part of our ongoing search to probe the stereoelectronic requirements for optimal trypanocidal activity, we present herein the synthesis and biology of the new secondary hydroxamic acids **5** and **6** (Fig. 1). Surprisingly, the trypanocidal activity results obtained for these compounds indicated a dramatic decrease in potency (∼2000-fold), compared to their nonmethylated counterparts **1a** and **2**, as shown in Table 1.

**Table 1:** Activity of acetohydroxamic acids **1a**, **2**, **5** and **6** tested against cultured bloodstream-form *T. brucei* (pH = 7.4) (see Supplementary data)

Compd	*T. brucei*	
	IC_50_^a^^,^^b^ (nM)	IC_90_^a^^,^^b^ (nM)
**1a**	90 ± 16 (79 ± 6)	155 ± 7 (148 ± 8)
**2**	17 ± 1 (18 ± 1)	26 ± 3 (24 ± 1)
**5**	246 × 10^3^ (106 × 10^3^)	523 × 10^3^ (198 × 10^3^)
**6**	37 × 10^3^ (35 × 10^3^)	47 × 10^3^ (45 × 10^3^)

a Concentrations required to inhibit growth of *T. brucei* by 50% and 90%, respectively. For the active compounds **1a** and **2**, IC_50_ and IC_90_ data are the mean of triplicate experiments ± SEM (standard error of the mean).

b IC_50_ and IC_90_ data for the respective hydrochlorides are shown in parentheses.

This vast difference in the trypanocidal action of the new analogues can probably be attributed to the predominance of different conformer(s). In order to verify this argument, we conducted a series of NMR experiments on hydroxamates **1a**, **2**, **5** and **6**, accompanied by theoretical calculations.

The synthesis of the new methylated hydroxamic acids **5** and **6** is shown in Scheme 1. Reaction of the carboxylic acids **7** and **8**, prepared as previously reported,^9^ with *O*-benzyl-*N*-methyl hydroxylamine in the presence of CDI in dry THF, led to the formation of the respective *O*-benzyl-*N*-methyl hydroxamates **9** and **10**. Subsequent *O*-benzyl deprotection, by hydrogenolysis, gave the corresponding methyl hydroxamic acid analogues **5** and **6** in high yields.

**Scheme 1:**
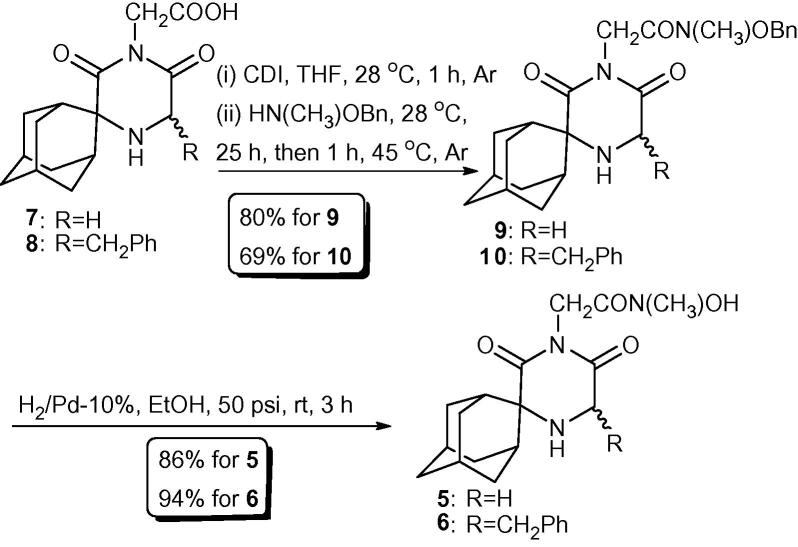
Synthesis of the new *N*-methyl hydroxamate analogues **5** and **6**.

The NMR experiments were performed in DMSO-*d*_6_ at ambient temperature. DMSO was the solvent of choice since it simulates the biological environment. A previous NMR study on simple monoalkylhydroxamic acids using DMSO-*d*_6_ as the solvent^4^ concluded that the *Z* isomer predominated.

Structure elucidation was performed for compounds **1a**, **2**, **5** and **6** using routine 1D ^1^H, ^13^C and 2D gCOSY, gHSQC, gHMBC and NOESY NMR techniques. Interestingly, compounds **1a** and **2** exhibited four distinct peaks for the NH and OH protons of the hydroxamate group, which correspond to the *E* and *Z* isomers. Moreover, two distinct ^13^C NMR resonances appeared for the tertiary carbon atoms of the hydroxamate carbonyl group attributed to each of the two isomers. On the other hand, compounds **5** and **6**, which bear a CON(CH_3_)OH moiety instead of the CONHOH group of **1a** and **2**, respectively, showed only one resonance for the OH proton and a single NCH_3_ signal. Furthermore, the ^13^C NMR spectra of compounds **5** and **6** showed a single resonance for each of the hydroxamate carbonyl and NCH_3_ carbons.

The ^1^H NMR spectra of compounds **2** and **6** are depicted in Figure 2 (downfield region), and their full ^1^H NMR spectra in Supplementary data. Full assignment tables of the ^1^H and ^13^C signals for the four analogues studied are also provided in Supplementary data.

**Figure 2:**
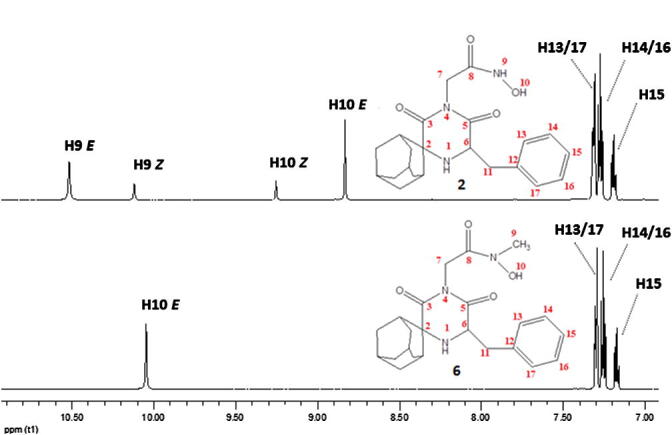
Downfield region of compounds **2** and **6** including proton assignments.

For compound **2**, the integration of the four signals from 8.83 to 10.52 ppm indicated that the two outermost peaks at 8.83 and 10.52 ppm are assigned to one isomer (isomer A), while the two inner ones, at 9.25 and 10.12 ppm, belong to isomer B, with an A:B ratio equal to 75:25. Furthermore, C8 of the hydroxamate carbonyl resonated at 164.1 ppm for the A isomer and at 169.5 ppm for the B isomer.

In order to determine the *E/Z* conformation, a 2D NOESY experiment was conducted. Figure 3 presents the expansion of the NOESY spectrum of compound **2**. Off diagonal signals due to the H7 methylene at 4.15 ppm are observed with two resonances for the major isomer (10.52 and 8.83 ppm) and one signal at 10.12 ppm for the minor isomer.

**Figure 3:**
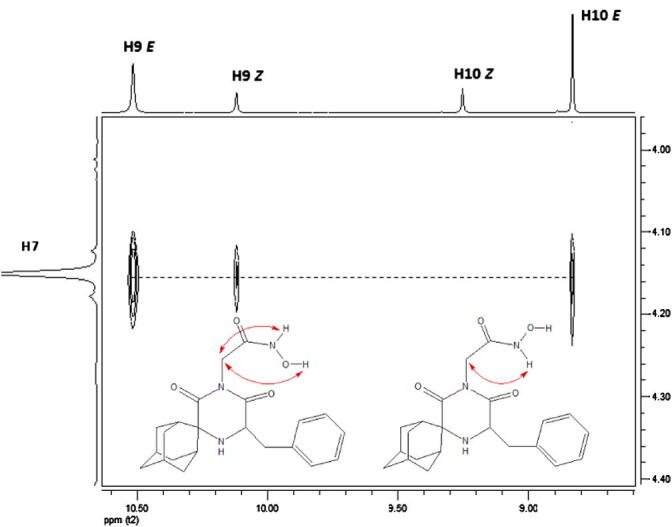
Expansion of the 2D NOESY spectrum of compound **2**.

From molecular modelling studies of the *E* and *Z* isomers of compound **2**, the distances between H9, H10 and H7 in both isomers have been calculated, and only H10 (OH) of the *Z* conformation is a long distance from H7 (∼5 Å), which may explain the absence of an NOE signal; thus H10 of the *Z* isomer is assigned to the signal at 9.25 ppm, and consequently H9 is assigned to that at 10.12 ppm.

As a result, the minor isomer is *Z* and the major is *E*. This is also in accordance with theoretical energy calculations of the *E* and *Z* isomers showing 66.3 kcal mol^−1^ for the *Z* and 65.4 kcal mol^−1^ for the *E* isomer. Finally, H9 and H10 of the *E* isomer are assigned to the signals at 10.52 and 8.83 ppm, respectively. The above assignments are in agreement with the 2D heteronuclear gHMBC spectrum, where a correlation between C8 and H9 is observed.

The same methodology was used for the assignment of the signals obtained for compound **6** (Supplementary data). Once again, an NOE signal was observed between H10 (OH) at 10.05 ppm and the H7 methylene, while the absence of an NOE between the NCH_3_ peak at 3.06 ppm and the methylene H7 protons, provided evidence for the *E* configuration of the molecule.

Systematic probing of the C7-C8-N-H9 dihedral angle of compound **2** produced two energy minima, one for the *E* conformation (58.4 kcal mol^−1^) and the other for the *Z* conformation (59.8 kcal mol^−1^, Fig. 4). Similarly, compound **6** provides one energy minimum for the *E* (58.8 kcal mol^−1^) and one for the *Z* (62.4 kcal mol^−1^). The energy barrier for the *E/Z* interconversion for compound **6** is higher than that of **2**, with an energy difference of ∼5 kcal mol^−1^ (Fig. 5).

**Figure 4:**
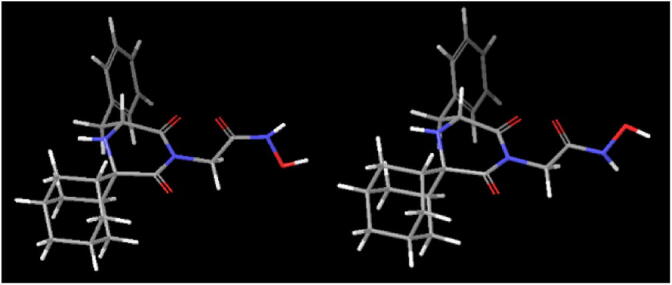
*E* (left) and *Z* (right) low energy conformations of bioactive compound **2**.

**Figure 5:**
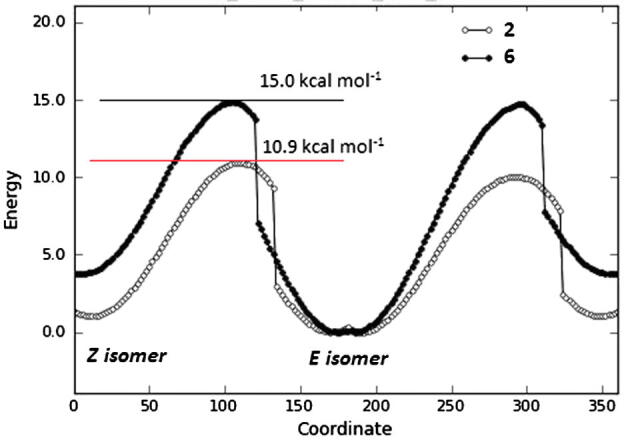
Relative energy versus coordinate plot from a systematic study of the CONH dihedral angle of the hydroxamate group.

In conclusion, NMR studies indicate that the primary hydroxamic acid derivatives **1a** and **2**, in their amide form, adopt preferentially the *E* conformation (*E*/*Z* = 75/25) in DMSO at ∼25 °C, whereas in their respective secondary *N*-methylated congeners **5** and **6**, the *E* conformer is the only one present in solution. These results are in accordance with in silico theoretical calculations.

Given that the *Z* conformation is a prerequisite for complexation with a metal ion, the failure of the methylated hydroxamic acid analogues **5** and **6** to produce a similar biological effect to their congeners **1a** and **2** might be attributed to the absence of the respective *Z* conformer in the binding site of the metalloenzyme. In the case of the active molecules **1a** and **2**, as the minor *Z* conformer is complexed to the metal ion in the catalytic site of the enzyme, the *E*/*Z* equilibrium is shifted to the active conformer () inducing metalloenzyme activity inhibition.

## Supplementary data

Supplementary data Experimental procedures and spectral data.
