# Supplementary material for: An E/Z conformational behaviour study on the trypanocidal action of lipophilic spiro carbocyclic 2,6-diketopiperazine-1-acetohydroxamic acids
Source: Tetrahedron Lett. 2013 Jun 19;54(25):3238–40. doi: 10.1016/j.tetlet.2013.03.128 (PMC3661977; doi:10.1016/j.tetlet.2013.03.128)
Supplement: Supplementary data — Experimental procedures and spectral data. [file mmc1.docx]

**Supplementary Data**

An *E*/*Z* conformational behaviour study on the trypanocidal action of lipophilic spiro carbocyclic 2,6-diketopiperazine-1-acetohydroxamic acids

Alexandra Tsatsaroni^a^, Grigoris Zoidis^a^, Panagiotis Zoumpoulakis^b^, Andrew Tsotinis^a^, Martin C. Taylor^c^, John M. Kelly^c^, George Fytas*^a^

^a^ Faculty of Pharmacy, Department of Pharmaceutical Chemistry, University of Athens, Panepistimioupoli-Zografou, GR-15771, Athens, Greece. Fax:+302107274747; Tel:+302107274810;

E-mail:gfytas@pharm.uoa.gr

^b^ Institute of Biology, Medicinal Chemistry and Biotechnology, National Hellenic Research Foundation, 48 Vas. Constantinou Ave., 11635, Athens, Greece

^c^ Department of Pathogen Molecular Biology, London School of Hygiene and Tropical Medicine, Keppel Street, London WC1E 7HT, UK

List of Contents

I Experimental. Chemistry…………………………………………………………………………...3

Ia General……………………………………………………………………………………………..3

Ib Experimental procedures for the synthesis of compounds 9, 10, 5 and 6, and their characterization data…………………………………………………………………………………4

IIa Structure elucidation of compounds 1a, 5, 2 and 6 (Tables S1-S4)…….….…...…………….7

IIb Methods for molecular modelling studies……………………………………………………….9

III Experimetal. Biological assays………………………………………………………………….10

IV Copies of NMR spectra………………………………………………………………………….11

^1^H NMR of 9………………………………………………………………………………………….12

^13^C NMR of 9…………………………………………………………………………………………13

DEPT NMR of 9…………………………………………………………………………….………..14

HSQC NMR of 9……………………………………………………………………………………..15

COSY NMR of 9……………………………………………………………………………………..16

^1^H NMR of 10…………………………………………….…………………………………………..17

^13^C NMR of 10………………………………………………………………………………………..18

DEPT NMR of 10…………………………………………………………………………………….19

HSQC NMR of 10………………………………………………………………………………….20

COSY NMR of 10………………………………………………………………………………….21

^1^H NMR of 2…………………………………………………………………...…………………..22

^13^C NMR of 2……………………………………………………….……………………………..23

gCOSY NMR of 2………………………………………………………………………….…...…24

NOESY NMR of 2……………………………………………………………………………..….25

NOESY NMR of 2 *(expansion)…*……………………………………………………………...…26

^1^H ^13^C gHSQC NMR of 2………………………………………………………………...…….….27

^1^H ^13^C gHMBC NMR of 2……………………………………………...……………………….…28

^1^H NMR of 6…………………………………………………………………….…………………29

^13^C NMR of 6…………………………………………………..………………………….……….30

gCOSY NMR of 6………………………………………………………………………………….31

NOESY NMR of 6………………………………………………………………………………….32

^1^H ^13^C gHSQC NMR of 6…………………………………………………………………………..33

^1^H NMR of 1a…………………………………………………………………………………...….34

^13^C NMR of 1a……………………………………………………………………………………...35

gCOSY NMR of 1a…………………………………………………………………………………36

NOESY NMR of 1a………………………………………………………………………………...37

^1^H ^13^C gHSQC NMR of 1a…………………………………………….…………………………...38

^1^H ^13^C gHMBC NMR of 1a………………………..……………………………………………….39

^1^H NMR of 5………………………………………………………………………………………..40

^13^C NMR of 5……………………………………………………………………………………….41

gCOSY NMR of 5………………………………………………………………………………….42

^1^H ^13^C gHSQC NMR of 5……………………………………….…………………………………43

^1^H ^13^C gHMBC NMR of 5…………………………………….…………………………….……..44

**I Experimental. Chemistry**

**Ia General**.

Melting points were determined using a Büchi capillary apparatus and are uncorrected. The ^1^H and ^13^C NMR spectra were obtained on either a Bruker MSL 400 (400 MHz ^1^H; 100 MHz ^13^C) or a Bruker AC200 (50 MHz ^13^C) spectrometer, using CDCl_3_ or DMSO-*d_6_* as solvent. Chemical shifts are reported in δ (ppm) with tetramethylsilane or solvent (DMSO-*d_6_*) as internal standard. Spliting paterns are designated as s, singlet; bs, broad singlet; d, doublet; dd, doublet of doublets; t, triplet; td, triplet of doublets; q, quartet; m, multiplet. Coupling constants (*J*) are expressed in units of Hertz (Hz). Carbon multiplicities were established by DEPT experiments. The 2D NMR experiments (HSQC and COSY) were performed for the elucidation of the structures of the newly synthesized compounds.

For the NMR conformational studies DMSO-*d_6_* and ultra precision NMR tubes (Norell 509-UP-7, 5mm) were used. Compounds were dissolved in DMSO-*d_6_* and a series of experiments were performed using a Varian 600 MHz spectrometer. All data were collected using pulse sequences and phase-cycling routines provided in the Varian libraries of pulse programs. Data processing including Fourier transformation, baseline correction, phasing, peak peaking and integrations were performed using MestReNova software v.6.0.1. The gCOSY, ^1^H-^13^C gHSQC and ^1^H-^13^C gHMBC experiments were performed with gradients.^10,11^ The NOESY experiment was recorded using standard pulse sequence in the phase-sensitive mode and was measured at 200 ms mixing time. The ^1^H sweep width was 7860 at 600 MHz.^12^ Typically, the homonuclear proton spectra were acquired with 4096 data points in t2, 16-64 scans, 256-512 increments in t1 and a relaxation delay of 1-1.5 s. The ^1^H-^13^C gHSQC spectrum was recorded with 1024 data points in t2, 32 scans per increment, 128 increments in t1 and a relaxation delay of 1s. The ^1^H-^13^C gHMBC spectrum was recorded with 4096 data points in t2, 64 scans per increment, 256 increments in t1 and a relaxation delay of 1s.^13,14^ The ^13^C spectral width was 30,000 and 36,000 Hz for the gHSQC and gHMBC experiments, respectively. Low resolution mass spectra (MS) were measured either by chemical ionization (CI) in positive mode using methane as CI reagent gas or by electron impact (EI) on a Thermo Electron Corporation DSQ mass spectrometer. High resolution mass spectra (HRMS) were performed on a Thermo LTQ-Orbitrap Velos spectrometer under electrospray ionization (ESI) in positive mode. Analytical thin-layer chromatography (TLC) was conducted on precoated Merck silica gel 60 F_254_ plates (layer thickness 0.2 mm) with the spots visualized by iodine vapors and/or UV light. Gravity and flash column chromatography purification were carried out on silica gel 60 (70-230 mesh) and (40-63 mesh), respectively. Elemental analyses (C, H, N) were within ±0.4% of the theoretical values. The commercial reagents were purchased from Alfa Aesar, Sigma-Aldrich, and Merck, and were used without further purification. The *O*-benzyl-*N-*methylhydroxylamine used, was prepared according to the literature procedure.^15^ Organic solvents used were in the highest purity, and when necessary, were dried by the standard methods. Solvent abbreviations: THF, tetrahydrofuran; Et_2_O, ethyl ether; MeOH, methanol; EtOH, ethanol; AcOEt, ethyl acetate; DMSO, dimethylsulfoxide.

Ib Experimental procedures for the synthesis of compounds 9, 10, 5 and 6, and their characterization data.

***N*-Methyl-3,5-dioxo-*N*-(phenylmethoxy)spiro[piperazine-2,2΄-tricyclo[3.3.1.1^3,7^]decane]-4-acetamide 9**

To a solution of the carboxylic acid **7**^9b^ (400 mg, 1.37 mmol) in dry THF (20 mL) 1,1΄-carbonyldiimidazole (266 mg, 1.64 mmol) was added, and the mixture was stirred at 28 °C for 1 h under argon. Then, a solution of *O*-benzyl-*N*-methylhydroxylamine (225 mg, 1.64 mmol) in THF (3 mL) was added, and stirring was continued at 28 °C for 24 h and then at 45 °C for 1 h under argon. After removal of the solvent *in vacuo*, water (35 mL) was added, and the mixture was extracted with ethyl acetate (3x35 mL). The combined extracts were washed with brine (2x35 mL), dried (Na_2_SO_4_) and evaporated under vaccum. The viscous oily residue was purified by column chromatography on silica gel eluting first with Et_2_O-*n*-hexane 1:1 and then with Et_2_O-AcOEt 1:1 to give the *O*-benzyl protected hydroxamate **9** as a white solid (450 mg, 80%): mp 113-115 °C (AcOEt/Et_2_O-*n*-pentane); ^1^H NMR (400 MHz, CDCl_3_) δ 1.54 (d, 2Η, *J* = 12.4 Hz, 4΄e, 9΄e-H), 1.68-1.79 (m, 5Η, 1, 6΄, 8΄e, 10΄e-H), 1.84 (s, 1Η, 7΄-H), 1.90 (s, 1Η, 5΄-H), 2.04 (s, 2Η, 1΄, 3΄-H), 2.32 (t, 4Η, *J* = 13.4 Hz, 4΄a, 8΄a, 9΄a, 10΄a-H), 3.19 (s, 3Η, C*H_3_*), 3.73 (s, 2Η, 6-H), 4.64 (s, 2Η, C*H_2_*CON(CH_3_)OCH_2_Ph), 4.94 (s, 2Η, CON(CH_3_)OC*H_2_*Ph), 7.39 (s, 5Η, aromatic Η); ^13^C NMR (100 MHz, CDCl_3_) δ 27.2 (5΄-C), 27.4 (7΄-C), 32.5 (4΄,9΄-C), 32.8 (1΄,3΄-C), 33.3 (8΄,10΄-C), 34.1 (*C*H_3_), 38.2 (6΄-C), 40.7 (*C*H_2_CON(CH_3_)OCH_2_Ph), 44.6 (6-C), 60.6 (2,2΄-C), 76.7 (CON(CH_3_)O*C*H_2_Ph), 128.9, 129.1, 129.2 (2, 3, 4, 5, 6-aromatic C), 134.4 (1-aromatic C), 168.7 (*C*ON(CH_3_)OCH_2_Ph), 172.6, 174.8 (3,5-C); CI^+^ MS: m/z 412.2 ([M+H]^+^, 59), 411.2 ([M]^+^, 50), 384.2 (42), 275.1 (28), 247.0 (86), 220.0 (44), 219.0 (85), 163.1 (100); Anal.Calcd for C_23_H_29_N_3_O_4_ : C, 67.13; H, 7.10; N, 10.21; Found : C, 67.08; H, 7.04; N, 10.12.

***N*-Hydroxy-*N*-methyl-3,5-dioxospiro[piperazine-2,2΄-tricyclo[3.3.1.1^3,7^]decane]-4-acetamide 5**

A solution of the *O*-benzyl hydroxamate **9** (850 mg, 2.06 mmol) in absolute EtOH (80 mL) was hydrogenated over Pd-C 10% (102 mg) for 3 h at room temperature and 50 psi. The catalyst was filtered off, washed with EtOH (3x15 mL), and the combined filtrates were evaporated *in vacuo*. The remaining pale yellow solid was purified by column chromatography on silica gel using AcOEt as eluent to afford the title compound **5** as a white solid (570 mg, 86%): mp 185-188 °C (dec) (AcOEt/Et_2_O-*n*-pentane); ^1^H NMR (400 MHz, DMSO-*d_6_*) δ 1.43 (d, 2Η, *J* = 12.0 Hz, 4΄e, 9΄e-H), 1.56-1.68 (m, 4Η, 6΄, 8΄e, 10΄e-H), 1.76 (s, 1Η, 7΄-H), 1.79 (s, 1Η, 5΄-H), 1.96 (s, 2Η, 1΄,3΄-Η), 2.20-2.33 (bs, 4Η, 4΄a, 8΄a, 9΄a, 10΄a-H), 3.10 (s, 3Η, C*H_3_*), 3.11 (t, 1H, *J* = 8.2 Hz, 1-H), 3.56 (d, 2H, *J* = 8.5 Hz, 6-H), 4.49 (s, 2H, C*H_2_*CON(CH_3_)OH), 10.06 (s, 1H, O*H*); ^13^C NMR (100 MHz, DMSO-d_6_) δ 26.7 (5΄-C), 26.9 (7΄-C), 31.9 (1΄,3΄-C), 32.0 (4΄,9΄-C), 32.8 (8΄,10΄-C), 35.9 (*C*H_3_), 39.9 (*C*H_2_CON(CH_3_)OH), 44.0 (6-C), 59.4 (2,2΄-C), 166.7 (*C*ON(CH_3_)OH), 172.2, 174.6 (3,5-C); EI MS: m/z 322.2 ([M+H]^+^, 6), 321.1 ([M]^+^, 37), 293.1 (100), 247.1 (25), 219.1 (56); Anal. Calcd for C_16_H_23_N_3_O_4_: C, 59.79; H, 7.21; N, 13.08; Found: C, 59.48; H, 7.14; N, 13.24. Hydrochloride (**5**.HCl): it was prepared by treating a solution of **5** in AcOEt with ethereal HCl under ice-cooling and was fully precipitated by adding Et_2_O. The white solid was collected by filtration, triturated with ether, and dried *in vacuo*. Mp: progressively decomposed from 175 °C; Anal. Calcd for C_16_H_24_ClN_3_O_4_: C, 53.70; H, 6.76; N, 11.74; Found: C, 53.87; H, 6.97; N, 11.60.

**(*R,S*)-*N*-Hydroxy-*N*-methyl-3,5-dioxo-6-(phenylmethyl)spiro[piperazine-2,2΄-tricyclo [3.3.1.1^3,7^]decane]-4-acetamide 6**

Carboxylic acid **8**^9a^ (700 mg, 1.83 mmol) was coupled with *O*-benzyl-*N*-methylhydroxylamine (302 mg, 2.20 mmol) in the presence of 1,1΄-carbonyldiimidazol (357 mg, 2.20 mmol) in dry THF (30 mL), following the same procedure described for the preparation of the *O*-benzyl hydroxamate **9**. The resulting oily residue was purified by flash column chromatography on silica gel, using AcOEt-Et_2_O 1:1 as eluent to afford a white foamy solid, which strongly binds the aforementioned solvents. Removal of the entrapped solvents upon drying at 62-64 °C under vacuum (10^-3^ mmHg) in an Abderhalden apparatus gave the corresponding *O*-benzyl hydroxamate precursor **10** as a glass solid (630 mg, 69%); ^1^H NMR (400 MHz, CDCl_3_) δ 1.3-1.44 (m, 2H, 4΄e, 9΄e-H), 1.48-1.84 (complex m, 10H, 1, 3΄, 4΄a, 5΄, 6΄, 7΄, 8΄, 10΄e-H), 1.96 (d, 1H, *J* = 12.8 Hz, 9΄a-H), 2.13 (s, 1H, 1΄-H), 2.85 (d, 1H, *J* = 12.4 Hz, 10΄a-H), 2.88-2.98 (q, AMX, A region, *J_AX_* = 8.4 Hz, *J_AM_* = 13.8 Hz, C*H_A_*H_M_Ph), 3.12 (s, 3H, C*H_3_*), 3.35 (dd, 1H, AMX, M region, *J_MX_* = 3.6 Hz, *J_AM_* = 13.8 Hz, CH_A_*H_M_*Ph), 3.78-3.87 (q, 1H, AMX, X region, *J_MX_* = 3.6 Hz, *J_AX_* = 8.4 Hz, 6-H_X_), 4.48-4.64 (q_AB_, 2H, *J* = 16.6 Hz, C*H_2_*CON(CH_3_)OCH_2_Ph), 4.83-4.95 (q_AB_, 2H, *J* = 10.4 Hz, CON(CH_3_)OC*H_2_*Ph), 7.13-7.38 (m, 10H, aromatic H); ^13^C NMR (50 MHz, CDCl_3_) δ 27.1, 27.2 (5΄,7΄-C), 30.7 (1΄-C), 31.5 (4΄-C), 32.6 (9΄-C), 33.4 (8΄-C), 34.1 (10΄-C, *C*H_3_), 35.1 (3΄-C), 38.2 (*C*H_2_Ph, 6΄-C), 40.9 (*C*H_2_CON(CH_3_)OCH_2_Ph), 54.4 (6-C), 61.0 (2,2΄-C), 76.8 (CON(CH_3_)O*C*H_2_Ph), 127.0, 128.7, 128.9, 129.1, 129.2, 129.3, 134.4, 137.2 (aromatic C), 168.7 (*C*ON(CH_3_)OCH_2_Ph), 173.8, 174.5 (3,5-C); CI^+^ MS: m/z 502.2 ([M+H]^+^, 57), 501.2 ([M]^+^, 22), 474.2 (8), 411.2 ([M+H-CH_2_Ph]^+^, 12), 410.1 ([M-CH_2_Ph]^+^, 49), 337.1 (22), 252.1 (30); HRMS (ESI): [M+Na]^+^ calcd for C_30_H_35_N_3_O_4_, 524.2525, found 524.2529.

Compound **10** (1.06 mg, 2.11 mmol) was subjected to hydrogenolysis as described for the preparation of **5** from **9**. The crude product was purified by chromatography on a silica gel column using AcOEt-*n*-hexane 2:1 as eluent to give the title compound **6** as a white foamy solid, which strongly binds the eluting solvents. Removal of the entrapped solvents as described above for **10** afforded **6** as a white crystalline solid (820 mg, 94%): mp 132-135 °C; ^1^H NMR (400 MHz, DMSO-*d_6_*) δ 1.30 (t, 2H, *J* = 9.7 Hz, 4΄e, 9΄e-H), 1.50-1.78 (complex m, 8H, 4΄a, 5΄, 6΄, 7΄, 8΄, 10΄e-H), 1.83 (s, 1H, 3΄-H), 2.07 (s, 1H, 1΄-H), 2.17 (d, 1H, *J* = 11.8 Hz, 9΄a-H), 2.71-2.85 (complex m, 3H, 1,10΄a-H, C*H_A_*H_M_Ph), 3.09 (s, 3H, C*H_3_*), 3.31 (dd, 1H, AMX, M region, *J_MX_* = 3 Hz*, J_AM_* = 13.8 Hz, CH_A_*H_M_*Ph), 3.73 (td, 1H, *J* = 3.3 Hz, 10.6 Hz, 6-H_X_), 4.46-4.56 (q_AB_, 2H, *J_AB_* = 16.7 Hz, C*H_2_*CON(CH_3_)OH), 7.15-7.35 (m, 5H, aromatic Η), 10.08 (s, 1H, O*H*); ^13^C NMR (100 MHz, DMSO-d_6_) δ 26.6 (5΄-C), 26.7 (7΄-C), 30.1 (1΄-C), 30.9 (4΄-C), 32.1 (9΄-C), 32.7 (8΄-C), 33.4 (10΄-C), 34.0 (3΄-C), 36.0 (*C*H_3_), 37.4 (*C*H_2_Ph), 37.7 (6΄-C), 40.3 (*C*H_2_CON(CH_3_)OH), 54.4 (6-C), 59.9 (2,2΄-C), 126.2, 128.0, 129.1 (2, 3, 4, 5, 6-aromatic C), 138.6 (1-aromatic C), 166.8 (*C*ON(CH_3_)OH), 173.8, 174.6 (3,5-C); EI MS: m/z 412.2 ([M+H]^+^, 3), 411.2 ([M]^+^, 7), 383.1 (13), 321.2 ([M+H-CH_2_Ph]^+^, 18), 320.0 ([M-CH_2_Ph]^+^, 100), 252.1 (41); HRMS (ESI): [M+H]^+^ calcd for C_23_H_29_N_3_O_4_, 412.2236, found 412.2227. Hydrochloride (**6**.HCl): it was prepared by treating an ether solution of **6** with ethereal HCl under ice-cooling. The white precipitate was collected by filtration, triturated with ether, and dried *in vacuo*. Mp: 203-206 °C (dec); Anal. Calcd for C_23_H_30_ClN_3_O_4_: C, 61.66; H, 6.75; N, 9.38; Found: C, 61.35; H, 6.46; N, 9.21.

IIa Structure elucidation of compounds 1a, 2, 5 and 6.

| **Table S1.** NMR Assignment of compound **1a** | | | | |
| --- | --- | --- | --- | --- |
| **Proton** | **Major conformation (*E*)** | | **Minor conformation (*Z*)** | |
|  | **^1^H (ppm)** | **^13^C (ppm)** | **^1^H (ppm)** | **^13^C (ppm)** |
| 1’ | 1.93 (bs) | 31.8 | 1.93 (bs) | 31.8 |
| 3’ | 1.93 (bs) | 31.8 | 1.93 (bs) | 31.8 |
| 4’e | 1.42 (d) | 31.9 | 1.42 (d) | 31.9 |
| 4’a | 2.25 (t) | 31.9 | 2.25 (t) | 31.9 |
| 5’ | 1.79 (s) | 26.6 | 1.79 (s) | 26.6 |
| 6’ | 1.60-1.64 (m) | 37.7 | 1.60-1.64 (m) | 37.7 |
| 7’ | 1.75 (s) | 26.9 | 1.75 (s) | 26.9 |
| 8’e | 1.60-1.64 (m) | 32.7 | 1.60-1.64 (m) | 32.7 |
| 8’a | 2.25 (t) | 32.7 | 2.25 (t) | 32.7 |
| 9’e | 1.42 (d) | 31.9 | 1.42 (d) | 31.9 |
| 9’a | 2.25 (t) | 31.9 | 2.25 (t) | 31.9 |
| 10’e | 1.60-1.64 (m) | 32.7 | 1.60-1.64 (m) | 32.7 |
| 10’a | 2.25 (t) | 32.7 | 2.25 (t) | 32.7 |
| 1 | 3.10 (t) | -- | 3.10 (t) | -- |
| 2,2΄ | -- | 59.5 | -- | 59.5 |
| 3 | -- | 174.5 | -- | 174.6 |
| 5 | -- | 172.1 | -- | 172.2 |
| 6 | 3.56 (d) | 44.0 | 3.56 (d) | 44.0 |
| 7 | 4.13 (s) | 39.1 | 4.44 (s) | 39.6 |
| 8 | -- | 164.1 | -- | 169.5 |
| 9 | 10.51 (s) | -- | 10.11 (s) | -- |
| 10 | 8.82 (s) | -- | 9.24 (s) | -- |

| **Table S2.** NMR Assignment of compound **5** | | |
| --- | --- | --- |
| **Proton** | **^1^H (ppm)** | **^13^C (ppm)** |
| 1’ | 1.95 (s) | 31.9 |
| 3’ | 1.95 (s) | 31.9 |
| 4’e | 1.42 (d) | 32.0 |
| 4’a | 2.24-2.27 (m) | 32.0 |
| 5’ | 1.78 (s) | 26.6 |
| 6’ | 1.60-1.64 (m) | 37.8 |
| 7’ | 1.75 (s) | 26.9 |
| 8’a | 2.24-2.27 (m) | 32.8 |
| 8’e | 1.60-1.64 (m) | 32.8 |
| 9’e | 1.42 (d) | 32.0 |
| 9’a | 2.24-2.27 (m) | 32.0 |
| 10’e | 1.60-1.64 (m) | 32.8 |
| 10’a | 2.24-2.27 (m) | 32.8 |
| 1 | 3.11 (t) | -- |
| 2, 2’ | -- | 59.4 |
| 3 | -- | 174.6 |
| 5 | -- | 172.2 |
| 6 | 3.56 (d) | 44.0 |
| 7 | 4.48 (s) | 39.9 |
| 8 | -- | 166.7 |
| 9 | 3.07 (s) | 35.9 |
| 10 | 10.05 (s) | -- |

| **Table S3.** NMR Assignment of compound **2** | | | | |
| --- | --- | --- | --- | --- |
| **Proton** | **Major conformation (*E*)** | | **Minor conformation (*Z*)** | |
|  | **^1^H (ppm)** | **^13^C (ppm)** | **^1^H (ppm)** | **^13^C (ppm)** |
| 1’ | 2.04 (s) | 29.8 | 2.04 (s) | 30.0 |
| 3’ | 1.81 (s) | 34.0 | 1.81 (s) | 33.9 |
| 4’e | 1.27 (d) | 30.8 | 1.27 (d) | 30.8 |
| 4’a | 1.55-1.68 (m) | 30.8 | 1.55-1.68 (m) | 30.8 |
| 5’ | 1.75 (s) | 26.4 | 1.75 (s) | 26.4 |
| 6’ | 1.55-1.68 (m) | 37.6 | 1.55-1.68 (m) | 37.6 |
| 7’ | 1.55-1.68 (m) | 26.6 | 1.55-1.68 (m) | 26.6 |
| 8’ | 1.55-1.68 (m) | 32.6 | 1.55-1.68 (m) | 32.6 |
| 9’e | 1.27 (d) | 32.0 | 1.27 (d) | 32.0 |
| 9’a | 2.17 (d) | 32.0 | 2.17 (d) | 32.0 |
| 10’e | 1.55-1.68 (m) | 33.2 | 1.55-1.68 (m) | 33.2 |
| 10’a | 2.71-2.82 (m) | 33.2 | 2.71-2.82 (m) | 33.2 |
| 1 | 2.78 (m) | -- | 2.78 (m) | -- |
| 2,2΄ | -- | 59.9 | -- | 59.8 |
| 3 | -- | 174.5 | -- | 174.6 |
| 5 | -- | 173.6 | -- | 173.7 |
| 6 | 3.72 (td) | 54.1 | 3.72 (td) | 54.2 |
| 7 | 4.15 (dd) | 39.2 | 4.44 (dd) | 39.6 |
| 8 | -- | 164.1 | -- | 169.5 |
| 9 | 10.52 (s) | -- | 10.12 (s) | -- |
| 10 | 8.83 (s) | -- | 9.25 (s) | -- |
| 11 | 2.73 , 3.29 | 37.3 | 2.73 , 3.29 | 37.3 |
| 12 | -- | 138.6 | -- | 138.6 |
| 13/17 | 7.31 (d) | 129.1 | 7.31 (d) | 129.1 |
| 14/16 | 7.27 (t) | 127.9 | 7.27 (t) | 127.9 |
| 15 | 7.19 (t) | 126.1 | 7.19 (t) | 126.1 |

| **Table S4.** NMR Assignment of compound **6** | | |
| --- | --- | --- |
| **Proton** | **^1^H (ppm)** | **^13^C (ppm)** |
| 1’ | 2.06 (s) | 30.1 |
| 2’ | -- | 59.5 |
| 3’ | 1.81 (s) | 34.0 |
| 4’e | 1.29 (t) | 31.0 |
| 4’a | 1.52-1.75 (m) | 31.0 |
| 5’ | 1.52-1.75 (m) | 26.7 |
| 6’ | 1.52-1.75 (m) | 37.6 |
| 7’ | 1.52-1.75 (m) | 26.9 |
| 8’ | 1.52-1.75 (m) | 32.8 |
| 9’e | 1.29 (t) | 32.2 |
| 9’a | 2.15 (d) | 32.2 |
| 10’e | 1.52-1.75 (m) | 33.4 |
| 10’a | 2.73-2.82 (m) | 33.4 |
| 1 | 2.73-2.82 (m) | -- |
| 6 | 3.72 (td) | 54.1 |
| 11a | 2.73-2.82 (m) | 37.4 |
| 11b | 3.29 (dd) | 37.4 |
| 12 | -- | 137.0 |
| 13/17 | 7.31 (d) | 127.6 |
| 14/16 | 7.27 (t) | 126.5 |
| 15 | 7.19 (t) | 124.7 |
| 3 | -- | 172.4 |
| 5 | -- | 171.6 |
| 7 | 4.50 (q) | 40.3 |
| 8 | -- | 164.6 |
| 9 | 3.08 (s) | 36.0 |
| 10 | 10.07 (s) | -- |

IIb Methods for molecular modelling studies

Computer calculations were performed with Schrödinger Suite 2012 molecular modelling package.^16^ Compounds **2** and **6** were initially minimized using Molecular Mechanics with OPLS_2005 force field and a dielectric constant (ε) equal to 45 simulating the DMSO environment of the NMR solvent. Minimization was performed with TNCG (Truncated Newton Conjugate Gradient) algorithm using 1000 iterations and an energy tolerance of 0.01 kcal/mol-1 Å -1, to reach a local minimum. Coordinate Scan (Macromodel) was implemented in order to explore the preferred torsion angles that correspond to the lowest energy conformers and energy barriers due to *E/Z* conformation of the hydroxamate group. This method initiates a coordinate scan search that generates conformations by varying specified torsion angles. Intervals of 5^o^ were applied for single bond rotation. During the Coordinate Scan procedures, OPLS_2005 Force Field with Dielectric Constant equal to 45 and normal cut-off were used for the potential parameters and PRCG algorithm with 1000 iterations and convergence threshold equal to 0.001 were used for the minimization of the produced conformers.

III Experimetal. Biological assays

**Trypanocidal assays**

Cultured bloodstream form *Trypanosoma brucei* (strain Lister 427) were maintained at 37^o^C in HMI-9 medium (Invitrogen) supplemented with 10% v/v fetal bovine serum (BioSera), penicillin/streptomycin (GibcoBRL) and β–mercaptoethanol (Sigma) in a 5% CO_2_ atmosphere^17^. Trypanocidal activity was assessed by growing parasites in the presence of various drug concentrations to determine the levels which inhibited growth by 50% (IC_50_) and 90% (IC_90_). Experiments were performed using a 96-well microtitre plate format, with parasites seeded at 2.5x10^4^ ml^-1^, and the plates incubated at 37°C for 2 days. 20 µl alamarBlue™ was then added to each well and the plates returned to 37°C overnight. Fluorescence was read in a Gemini Fluorimeter at λ_ex_ 530 nm and λ_em_ 585 nm with a cut-off set at 570 nm (Molecular Devices).

10. Rance, M.; Sorensen, O. W.; Wagmer, G.; Ernst, R. R.; Wuthrich, K. *Biophys. Res. Commun*. **1983**, *117*, 479-485.

11. Bodenhausen, G.; Ruben, D. J. *Chem. Phys. Lett*. **1980**, *69*, 185-189.

12. Jeener, J.; Meier, B. H.; Bachmann, P.; Ernst, R. R. *J. Chem. Phys.* **1979**, *71*, 4546-4553.

13. Bax, A.; Summers, M. F. *J. Am. Chem. Soc.* **1986**, *108*, 2093-2094.

14. Bermel, W.; Wagner, K.; Griesinger, C. J. *Magn. Reson.* **1989**, *83*, 223-232.

15. Sharma, S. K.; Miller, M. J.; Payne, S. M. *J. Med. Chem.* **1989**, *32*, 357-367.

16. Maestro, version 9.2, 2011, Schrödinger, LLC, New York.

17. Hirumi, H.; Hirumi, K. *J Parasitol,* **1989**, *75*, 985-989.

**IV Copies of NMR spectra**

**^1^H NMR of *9* (400 MHz, CDCl_3_)**

***
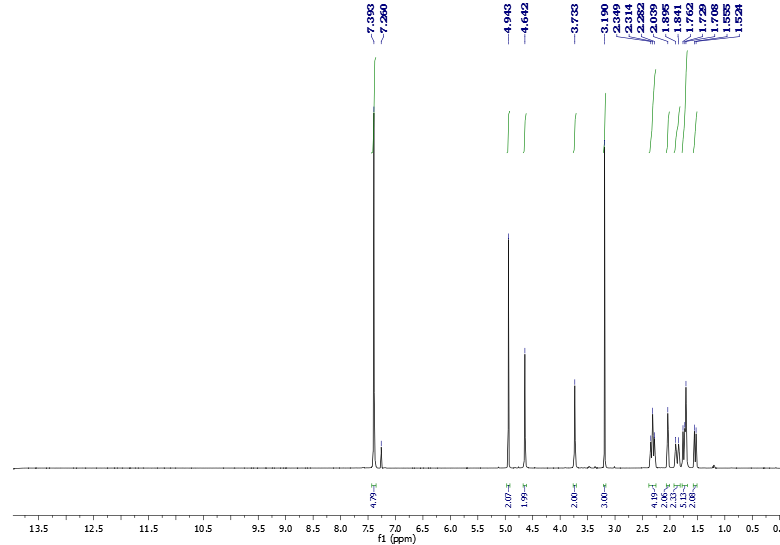
***

**^13^C NMR of *9* (100 MHz, CDCl_3_)**


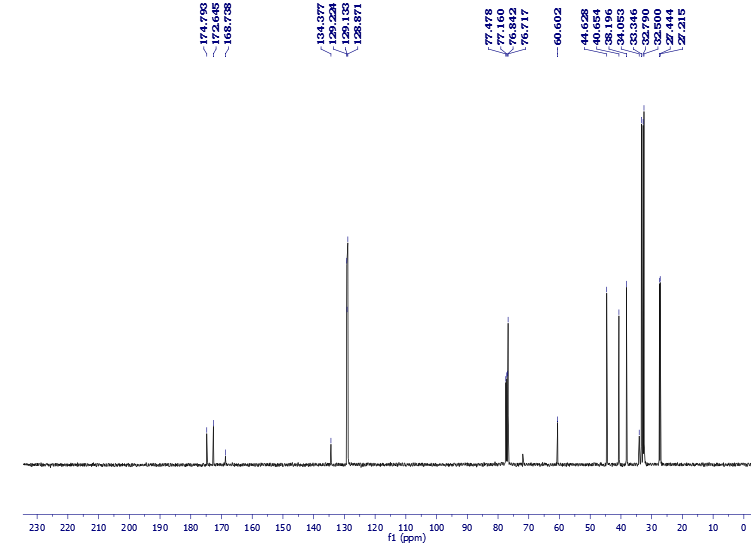


**DEPT NMR of *9* (100 MHz, CDCl_3_)**

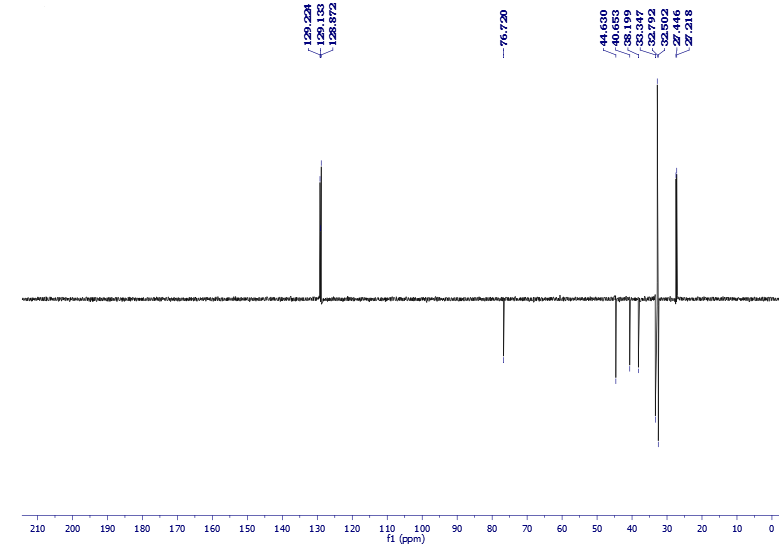


**HSQC NMR of *9* (400 MHz, CDCl_3_)**


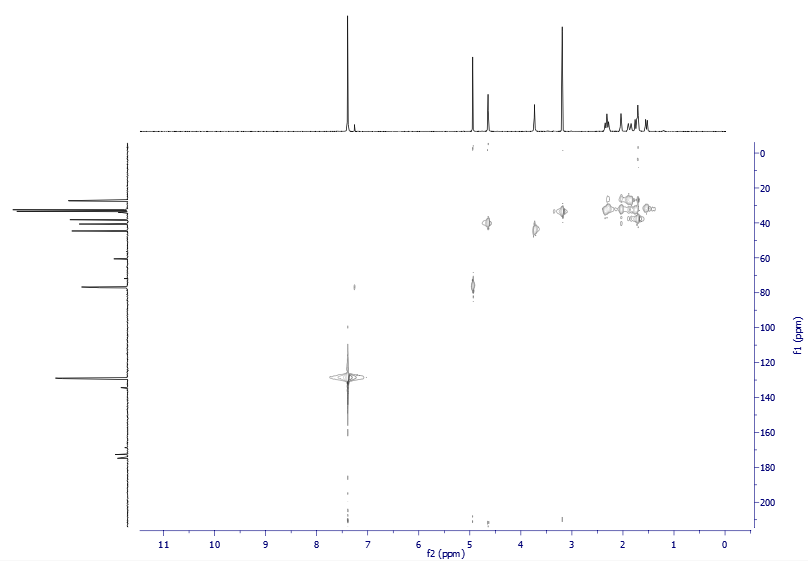


**COSY NMR of *9* (400 MHz, CDCl_3_)**


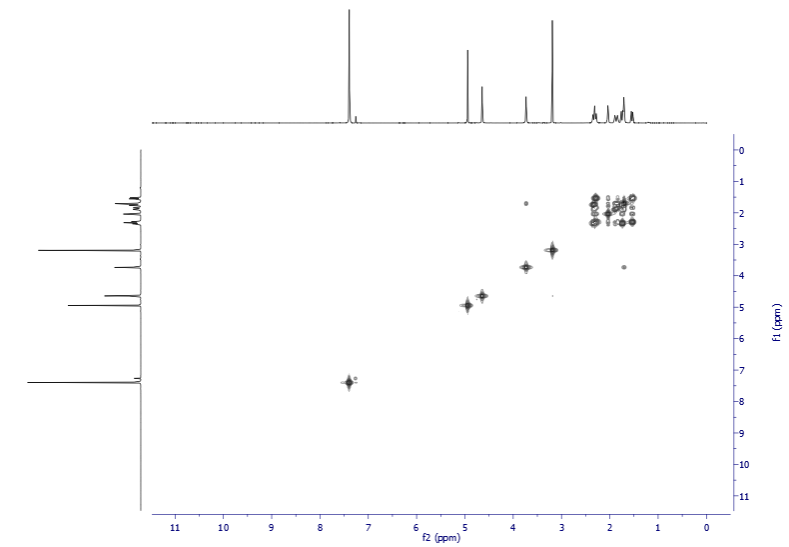


**^1^H NMR of *10* (400 MHz, CDCl_3_)**


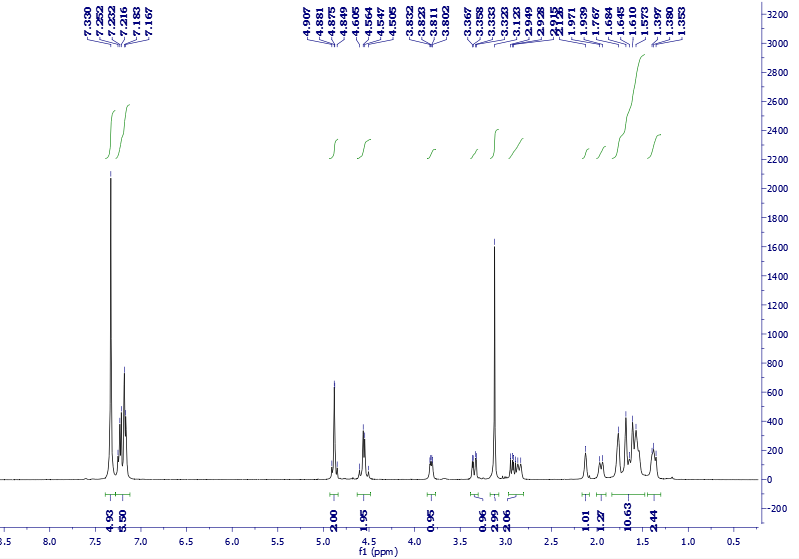


**^13^C NMR of *10* (50 MHz, CDCl_3_)**


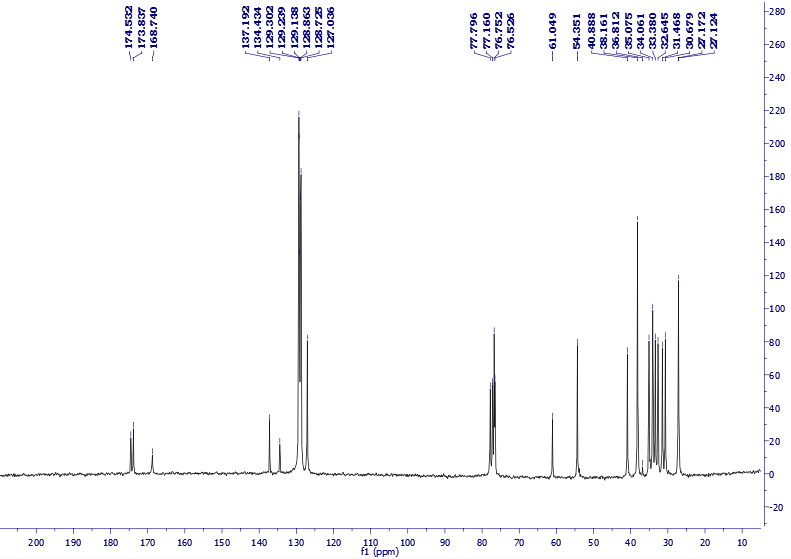


**DEPT NMR of *10* (50 MHz, CDCl_3_)**


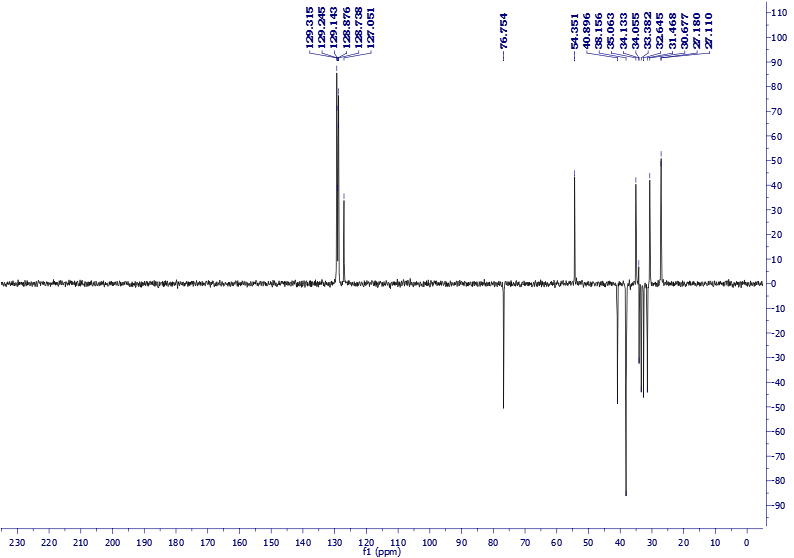


**HSQC NMR of *10* (400 MHz, CDCl_3_)**


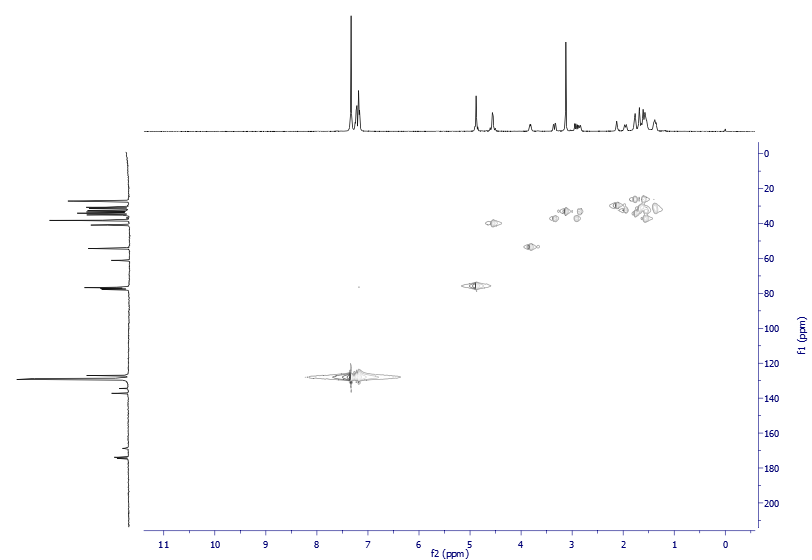


**COSY NMR of *10* (400 MHz, CDCl_3_)**

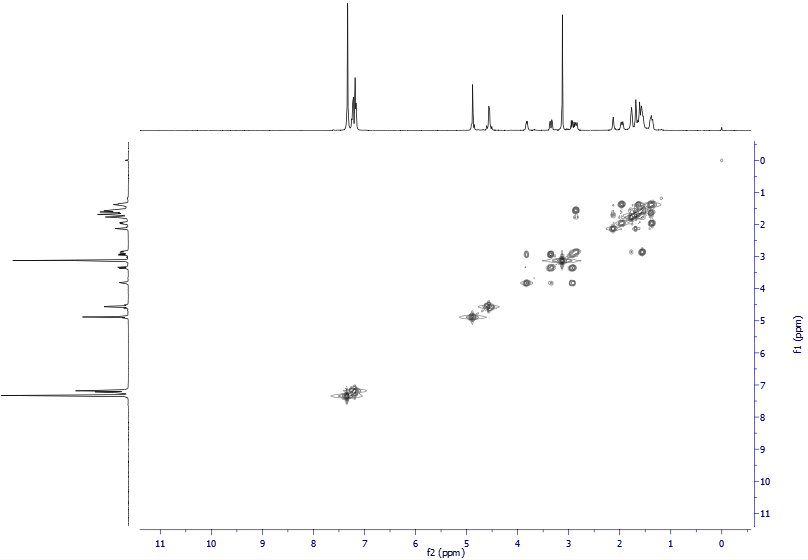


**^1^H NMR of *2* (600 MHz, DMSO-*d_6_*)**

**^13^C NMR of *2* (150 MHz, DMSO-*d_6_*)**


**gCOSY NMR of *2* (600 MHz, DMSO-*d_6_*)**


**NOESY NMR of *2* (600 MHz, DMSO-*d_6_*)**

**NOESY NMR of *2* (600 MHz, DMSO-*d_6_^­^*^)^ *(expansion)***


 **^1^H ^13^C gHSQC NMR of *2* (600 MHz, DMSO-*d_6_*)**


**^1^H ^13^C gHMBC NMR of *2* (600 MHz, DMSO-*d_6_*)**


**^1^H NMR of *6* (600 MHz, DMSO-*d_6_*)**

**^13^C NMR of *6* (150 MHz, DMSO-*d_6_*)**

**gCOSY NMR of *6* (600 MHz, DMSO-*d_6_*)**

**NOESY NMR of *6* (600 MHz, DMSO-*d_6_*)**


**^1^H ^13^C gHSQC NMR of *6* (600 MHz, DMSO-*d_6_*)**

**^1^H NMR of *1a* (600 MHz, DMSO-*d_6_*)**

**^13^C NMR of *1a* (150 MHz, DMSO-*d_6_*)**


**gCOSY NMR of *1a* (600 MHz, DMSO-*d_6_*)**

**NOESY NMR of *1a* (600 MHz, DMSO-*d_6_*)**

**^1^H ^13^C gHSQC NMR of *1a* (600 MHz, DMSO-*d_6_*)**

**^1^H ^13^C gHMBC NMR of *1a* (600 MHz, DMSO-*d_6_*)**

**^1^H NMR of *5* (600 MHz, DMSO-*d_6_*)**

**^13^C NMR of *5* (150 MHz, DMSO-*d_6_*)**

**gCOSY NMR of *5* (600 MHz, DMSO-*d_6_*)**

**^1^H ^13^C gHSQC NMR of *5* (400 MHz, DMSO-*d_6_*)**

0

1

2

3

4

5

6

7

8

9

10

11

f2 (ppm)

0

20

40

60

80

100

120

140

160

180

200

f

1

(

p

p

m

)

-3.75

-6.89

-2.20

-1.42

-1.04

-0.67

1.04

1.42

2.20

3.75

6.89

x

1

0

**^1^H ^13^C gHMBC NMR of *5* (600 MHz, DMSO-*d_6_*)**
